# Preoperative anterior coverage of the medial acetabulum can predict postoperative anterior coverage and range of motion after periacetabular osteotomy: a cohort study

**DOI:** 10.1186/s13018-020-01818-z

**Published:** 2020-08-10

**Authors:** Shinya Hayashi, Shingo Hashimoto, Tomoyuki Matsumoto, Koji Takayama, Tomoyuki Kamenaga, Takahiro Niikura, Ryosuke Kuroda

**Affiliations:** Department of Orthopaedic Surgery, Kobe University Graduate School of Medicine, 7-5-1 Kusunoki-cho, Chuo-ku, Kobe, Japan

**Keywords:** Periacetabular osteotomy, Range of motion, Anterior coverage

## Abstract

**Background:**

We hypothesized that preoperative pelvic morphology may affect postoperative anterior coverage and postoperative clinical range of motion (ROM) leading to postoperative pincer type femoroacetabular impingement (FAI). The aim of this study was to evaluate the relationships between preoperative bone morphology and postoperative ROMs to prevent postoperative FAI after periacetabular osteotomy.

**Methods:**

Sixty-eight patients (71 hips) with hip dysplasia participated in this study and underwent curved PAO. The acetabular fragment was usually moved only by lateral rotation of the acetabulum, without intraoperative anterior or posterior rotation. The pre- and postoperative three-dimensional center-edge (CE) angles were measured and compared to the postoperative ROM.

**Results:**

Preoperative medial anterior CE angle was significantly associated with postoperative anterior CE angle, and the correlation coefficient of medial anterior CE and postoperative anterior CE was higher than the coefficient of preoperative anterior CE and postoperative anterior CE (preoperative anterior CE, rr = 0.27, *p* = 0.020; preoperative medial anterior CE, rr = 0.54, *p* < 0.001). Femoral anteversion correlated with postoperative internal rotation angle at 90° flexion (*r* = 0.32, *p* = 0.021). In multiple linear regressions, postoperative internal rotation angle at 90° flexion angle was significantly affected by both medial CE angle through the medial one fourth of femoral head and femoral anteversion.

**Conclusions:**

Preoperative medial anterior acetabular coverage was associated with postoperative anterior acetabular coverage. Further, the combination with preoperative medial anterior acetabular coverage and femoral anteversion can predict postoperative internal rotation at 90° flexion. Therefore, the direction of acetabular reorientation should be carefully considered when the patients have high preoperative medial anterior CE angle and small femoral anteversion.

## Background

Developmental dysplasia of the hip (DDH) is the most common cause of secondary hip osteoarthritis (OA) in Japan; more than 70% of cases of hip OA are caused by DDH [1]. Therefore, many types of acetabular osteotomies have been developed to prevent OA [2–5]. The acetabular fragment is moved laterally, anteriorly, or in both directions to obtain femoral head coverage, and it has been suggested that both lateral and anterior rotations are more effective than lateral rotation alone to reduce contact pressure [6]. The movement of the acetabulum causes a mismatch between the acetabulum and femoral neck, which reduces flexion and internal rotation of hip range of motion (ROM) and can lead to femoroacetabular impingement (FAI) after periacetabular osteotomy (PAO) [7]. Several reports have described the over-coverage of the anterior acetabulum [8, 9]. Proper acetabular reorientation is essential to avoid FAI after PAO, although the most important purpose of acetabular osteotomy is reorienting the acetabulum into a normal position [10]. Suh et al. reported that only lateral rotation of the osteotomized acetabular fragments improved anterior coverage as well as lateral coverage [11]. Hamada et al. also reported, in a three-dimensional (3D)-computed tomography (CT) simulation study, that only lateral rotation of the acetabulum to achieve a lateral center-edge (CE) angle of 30° resulted in larger anterior coverage than that of normal hips in half of the DDH cases, and a wide variation of anterior coverage was seen after lateral rotation of the acetabulum [12]. We also demonstrated, in a 3D-simulation study, that anterior coverage was increased by only lateral rotation of the acetabulum without anterior or posterior rotation during curved PAO [13].

Several studies have demonstrated that computer-assisted ROM measurements using 3D models of the pelvis and femur can be used to assess the relative movement between the two segments up to the point of impingement and identify the impingement site [10, 14–17]. We also demonstrated, in a 3D-simulation study, that preoperative pelvic morphology was associated with postoperative anterior coverage and ROM [13].

However, these studies used simulated models and did not consider clinical ROM measurements. Several factors, especially those related to extra-articular structures, such as soft tissue contractures, may affect hip ROM after PAO. We recently discovered that postoperative excessive anterior acetabular coverage decreased clinical ROM after PAO [18].

We hypothesized that preoperative pelvic morphology may affect postoperative anterior coverage and postoperative clinical ROM. In this study, we aimed to evaluate the relationships among preoperative pelvic morphology medial to the femoral head center, postoperative acetabular anterior coverage at the femoral head center, and clinical ROM after PAO. Accordingly, we measured the 3D alignment of the pre- and postoperative acetabular coverage angles and compared these with postoperative ROMs.

## Methods

### Study participants and surgical procedure

Seventy-five patients who underwent curved PAO (CPO) for DDH from January 2015 to April 2018 were selected. Four patients did not complete the postoperative follow-up. Patients with femoral osteotomy and osteo-chondroplasty were excluded to avoid ROM bias. Data from 71 hips, contributed by 68 patients (62 women and 6 men), were included in the analysis. Preoperatively, all patients were classified as having grade 0 or 1 OA according to the Tönnis classification [19]. We also classified the patients for seriousness of DDH according to Severin classification system [20]. Of 68 patients, 11 patients were classified as Severin class Ib, 55 patients were class II, and 2 patients were class III. Average age at surgery was 33.4 years (range, 16–50 years). All patients underwent preoperative 3D planning with a 100-mm radius sphere using a navigation software (OrthoMap 3D Navigation System; Stryker Orthopaedics, Mahwah, NJ, USA). The CPO procedure was performed based on our previous study [21], and the acetabular fragment was usually moved only by lateral rotation of the acetabulum without anterior or posterior rotation during surgery.

### Clinical evaluation

Hip function was evaluated using two grading methods: first, the Japanese Orthopaedic Association (JOA) score, which allocates 40 points for pain, 20 points for ROM, 20 points for walking ability, and 20 points for activities of daily living, with a maximum total score of 100 points [22], and second, the University of California Los Angeles (UCLA) activity score, which describes subjects’ level of activity from 1 (“no physical activity, dependent on others”) to 10 (“regular participation in impact sports”). The JOA and UCLA scores were evaluated preoperatively and at 1-year postoperatively.

### Imaging evaluation

**Fig. 1:**
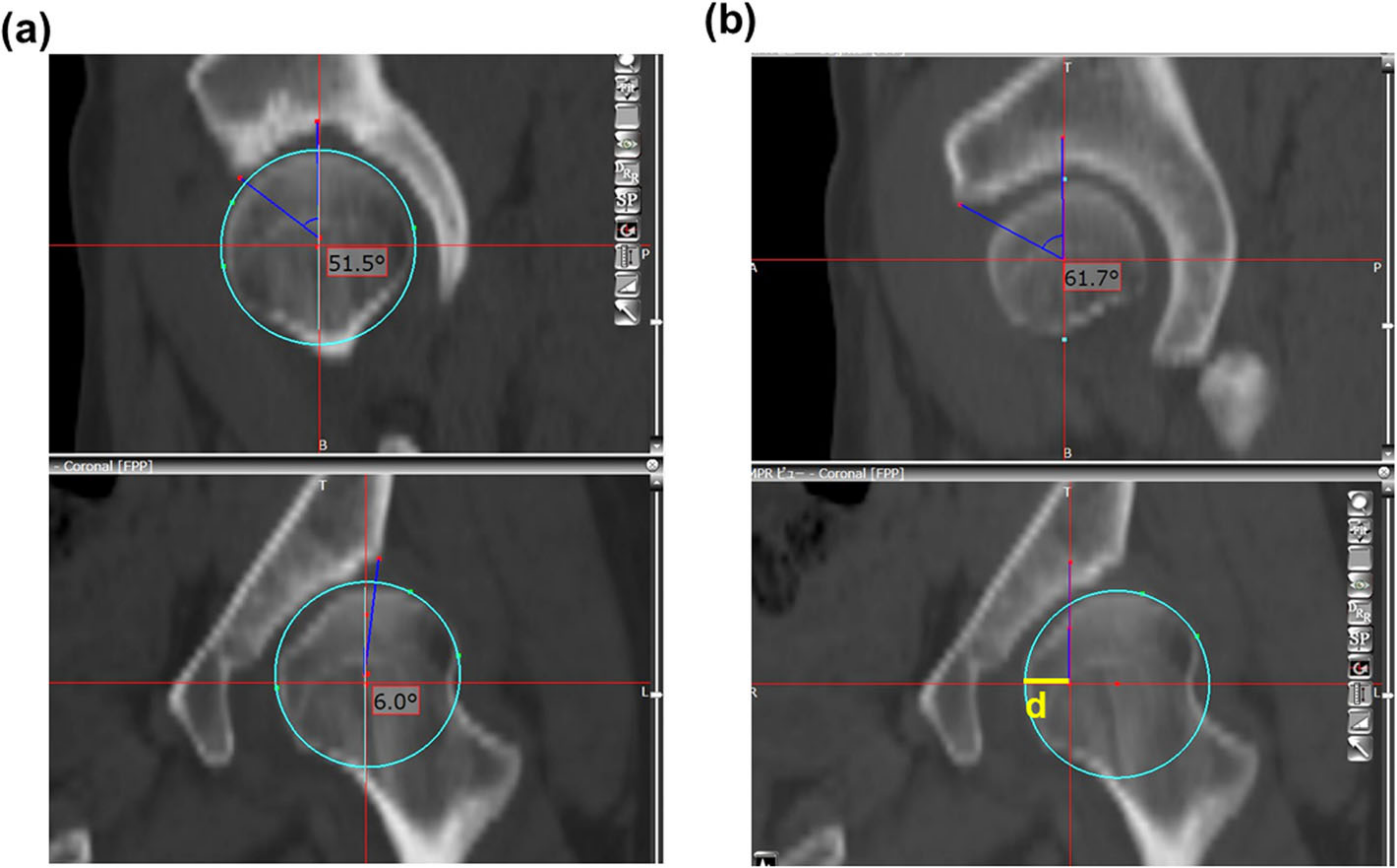
The center-edge angles. Photograph of the 3D template software (Zed Hip) to measure **a** preoperative lateral (6.0°, lower panel) and anterior (51.5°, upper panel) center-edge angles, and **b** coronal view through the femoral head center creating the sagittal plane through the medial one fourth of the femoral head. *d* = 1/4 × femoral head diameter (lower panel). Measurement of anterior center-edge angle on sagittal view through the medial one fourth of the femoral head (61.7°, upper panel)

Patients were positioned on the CT table in the supine position, and preoperative CT scans were performed from the pelvis to the knee joint using a 64-row multi-slice CT system at our hospital; the obtained image datasets were transferred to a 3D template software (Zed Hip; Lexi, Tokyo, Japan). The software operating window comprised of three multiplanar reformation viewers in the coronal, sagittal, and axial planes. The pelvic plane axis was defined according to the functional pelvic plane. The lateral CE angle and anterior CE angle were measured from the coronal and sagittal views through the femoral head center to quantitatively evaluate acetabular coverage in multiple directions (Fig. 1a). In addition, we measured the anterior CE angle through the medial quarter of the femoral head as an index reflecting the pelvic morphology in the sagittal plane, medial to the femoral head center (Fig. 1b). We measured the anterior CE angle at one fourth of the medial side of the femoral head radius on the coronal view through the center of the femoral head as the medial anterior CE angle (Fig. 1b). In detail, we made the approximate sphere of femoral head according to the femoral head center on coronal view and drew the horizontal line through the femoral head center (lower panel). The yellow line d means 1/4 diameter of femoral head. Upper panel of Fig. 1b shows sagittal plane view through the medial one fourth of the femoral head (lateral edge of line d). Finally, anterior center-edge angle on sagittal view through the medial one fourth of the femoral head was measured (e.g., 61.7°, upper panel of Fig. 1b).

Anatomical femoral anteversion angles were also measured with respect to the posterior condylar line axis of the femur [23].

### Statistical analysis

**Fig. 2:**
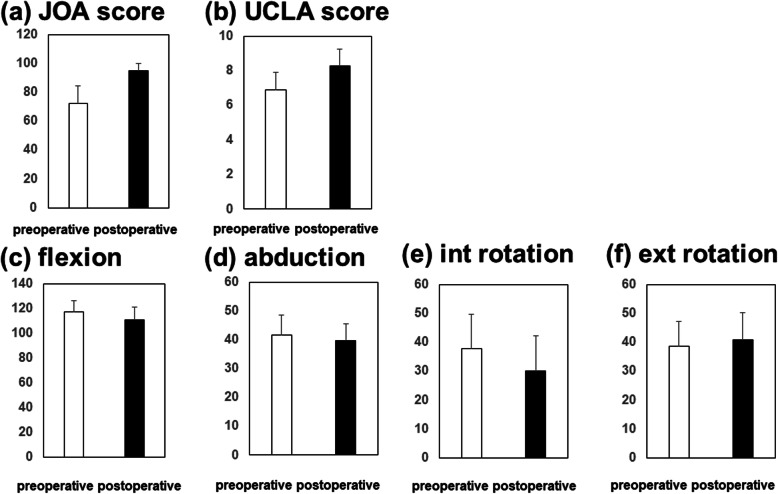
Clinical outcomes preoperatively and at 1 year postoperatively. **a** JOA score. **b** UCLA score. ROM of **c** flexion, **d** abduction, **e** internal rotation, and **f** external rotation

Pre- and postoperative lateral and anterior CE angles, JOA and UCLA scores, and hip ROM were compared using a Mann-Whitney *U* test (Fig. 2, Table 1). Correlations among pre- and postoperative CE angles, the medial anterior CE angle, and hip ROM (Tables 2 and 3) were analyzed using Pearson’s correlation coefficient. Multiple linear regression analyses were also performed with the results of internal rotation angle at 90° flexion as objective variable and the anterior CE angle through the medial one fourth of femoral head and femoral anteversion as explanatory variables (Table 4).

**Table 1:** Radiographic results

	Preoperative (°)	Postoperative (°)	*p* value
**Lateral CE**	11.9 ± 9.1	30.0 ± 8.8	< 0.001
**Anterior CE**	33.0 ± 15.5	52.6 ± 13.4	< 0.001
**Medial anterior CE**	49.7 ± 14.9

**Table 2:** Relation between preoperative and postoperative acetabular coverage

		Pre-lateral CE	Pre-anterior CE	Medial anterior CE
**Post-lateral CE**	Correlation	0.12	0.17	− 0.02
	*p* value	0.340	0.164	0.891
**Post-anterior CE**	Correlation	− 0.17	0.14	**0.57**
	*p* value	0.166	0.270	**< 0.001**

**Table 3:** Relation between preoperative center-edge angles or femoral anteversion and postoperative ROMs

		Post flexion	Post abduction	Post internal rotation	Post external rotation
**Preoperative anterior CE**	Correlation	− 0.13	− 0.13	− 0.09	− 0.14
	*p* value	0.28	0.309	0.447	0.269
**Medial anterior CE**	Correlation	**− 0.31**	− 0.09	**− 0.44**	0.13
	*p* value	**0.014**	0.47	**< 0.001**	0.34
**Femoral anteversion**	Correlation	0.03	0.01	**0.31**	− 0.57
	*p* value	0.821	0.960	**0.021**	**< 0.001**

**Table 4:** Multiple linear regression analysis

Explanatory variables	Internal rotation at 90° flexion		
	*r*	Standardized *r*	*p* value
Medial anterior CE	− 0.28	− 0.35	< 0.001
Femoral anteversion	0.24	0.27	0.004

All figure data are expressed as mean ± standard deviation unless otherwise indicated. The data were analyzed using SPSS version 16.0 software (IBM Corp., Armonk, NY, USA). *p* values < 0.05 were considered statistically significant.

## Results

### Radiographical and clinical outcomes

The radiographical outcomes are shown in Table 1. The mean values of pre- and postoperative lateral CE angles were 11.9° ± 9.1 and 30.0° ± 8.8, respectively (*p* < 0.001), and the mean pre- and postoperative anterior CE angles were 43.0° ± 15.5 and 62.6° ± 13.4, respectively (*p* < 0.001). Both lateral and anterior CE angles significantly increased pre- and postoperatively (Table 1).

Clinical evaluations are shown in Fig. 2. Mean values of postoperative JOA and UCLA activity scores were 95.1 ± 5.2 points and 8.3 ± 1.2 points, respectively, both of which showed significant improvement postoperatively (Fig. 2).

The mean pre- and postoperative ROM was 117° and 111° for flexion (*p* < 0.001), 42° and 40° for abduction (*p* = 0.042), 38° and 30° for internal rotation at 90° of hip flexion (*p* < 0.001), and 39° and 41° for external rotation with leg extension (*p* = 0.253). Flexion, abduction, and internal rotation ROM were significantly decreased postoperatively (Fig. 2).

### Preoperative medial anterior acetabular coverage was strongly correlated with postoperative anterior acetabular coverage

The correlation between pre- and postoperative acetabular coverage was evaluated. We noted significant associations between preoperative lateral or anterior CE angles and postoperative lateral or anterior CE angles (Table 2). The preoperative medial anterior CE angle was significantly associated with the postoperative anterior CE angle, and the correlation coefficient of the medial anterior CE and postoperative anterior CE was higher than the coefficient of the preoperative anterior CE and postoperative anterior CE (Table 2).

### Correlation coefficients between morphology parameters and ROMs

Table 3 provides a summary of the results of the correlation analysis between morphology parameters and simulated ROMs (flexion, extension, external rotation, and internal rotation). Preoperative anterior CE angle did not show any significant correlation with ROMs after CPO, but preoperative anterior CE angle through medial one fourth showed a significant correlation with flexion (*r* = − 0.31, *p* = 0.014) and internal rotation at 90° flexion (*r* = − 0.44, *p* < 0.001) (Table 3). Femoral anteversion showed a significant correlation with internal rotation angle at 90° flexion (*r* = 0.32, *p* = 0.021) and external rotation (*r* = − 0.57, *p* < 0.001) (Table 3). In multiple linear regression, internal rotation angle at 90° flexion angle was significantly affected by both medial CE angle through the medial one fourth of femoral head and femoral anteversion (Table 4).

## Discussion

Patients with DDH display smaller CE angles and a more anteverted femoral neck [24, 25]. A 3D analysis by Nakahara et al. showed that acetabular coverage in DDH was significantly lower, but the wave-shaped section of the rim was similar to that of normal hips, although the acetabulum is shallower overall, and those morphological differences affect ROM [26]. Maximum internal rotation at 90° of flexion in DDH was significantly larger when compared to normal hip joints [26]. Hamada et al. reported in a computer simulation study that rotational acetabular osteotomy (RAO) surgery increased both lateral and anterior acetabular coverage and decreased the ROM of flexion and internal rotation at 90° flexion after RAO [12]. We previously reported that the anterior acetabular coverage after simulated PAO was associated with parameters reflecting the pelvic morphology on a sagittal plane medial to the femoral head center, and the maximal flexion and internal rotation angle obtained by ROM simulation after simulated PAO were also significantly associated with these parameters [13]. Our results demonstrated that preoperative medial anterior acetabular coverage was associated with postoperative anterior acetabular coverage and postoperative clinical ROMs of flexion and internal rotation.

A previous computer simulation study demonstrated that the average impingement free ROM in flexion was 130° and in internal rotation was 50° in normal healthy subjects [12]. The patients suffering DDH and osteoarthritis may need less ROMs in daily activity because of less activity. One hundred and ten degrees of flexion, 40° of abduction, and 30° of internal rotation are enough for daily activities of DDH patients as per previous reports [12, 26]. We demonstrated a significant association between the preoperative medial anterior CE angle, femoral anteversion and postoperative ROM in internal rotation by multiple linear regression analysis and added an approximate formula; postoperative internal rotation = − 0.35 × medial anterior CE + 0.27 × femoral anteversion. Based on our result, we can predict postoperative ROM of internal rotation before surgery using this formula. Surgeons need to pay attention to the movement direction of the acetabular fragment in patients with a high medial anterior CE and small femoral anteversion. On the other hand, better coverage could improve length of function for the hip. The physician should inform the patient to avoid exhausting and demanding exercises after this surgery. Better joint congruency can be achieved with PAO so more stable and length joints are possible.

There are limitations to this study. The cohort was not large enough to enable a full evaluation of clinical ROM and the acetabular reorientation angle. Second, ROM is not only affected by the acetabular fragment and femur version. ROM can be influenced by pain, scar tissue, and labral irritability. Further investigation into these factors is required. Third, the study did not compare acetabular coverage angle and postoperative QOL other than ROMs. We need an assessment of QOL after PAO with lateral rotation and comparison with the outcomes of lateral and anterior rotation in the further study.

## Conclusions

In conclusion, preoperative medial anterior acetabular coverage was associated with postoperative anterior acetabular coverage and postoperative ROM in flexion and internal rotation; however, the ROM can be influenced by multiple factors. Therefore, the direction of acetabular reorientation should be carefully considered in patients with a high medial anterior CE and small femoral anteversion. The surgeon must analyze femoral anteversion and medial anterior CE prior to performing periacetabular osteotomy.

## Data Availability

All data generated or analyzed during this study are included in this published article.

## Abbreviations

DDH: Developmental dysplasia of the hip
OA: Osteoarthritis
ROM: Range of motion
FAI: Femoroacetabular impingement
PAO: Periacetabular osteotomy
3D: Three-dimensional
CT: Computed tomography
CE: Center-edge
CPO: Curved periacetabular osteotomy
JOA: Japanese Orthopaedic Association
UCLA: University of California Los Angeles
RAO: Rotational acetabular osteotomy
QOL: Quality of life
